# Circulating platelets as liquid biopsy sources for cancer detection

**DOI:** 10.1002/1878-0261.12859

**Published:** 2020-12-14

**Authors:** Mafalda Antunes‐Ferreira, Danijela Koppers‐Lalic, Thomas Würdinger

**Affiliations:** ^1^ Department of Neurosurgery Cancer Center Amsterdam Amsterdam University Medical Centers VU University Medical Center Amsterdam The Netherlands

**Keywords:** cancer, diagnostics, liquid biopsy, platelets, RNA

## Abstract

Nucleic acids and proteins are shed into the bloodstream by tumor cells and can be exploited as biomarkers for the detection of cancer. In addition, cancer detection biomarkers can also be nontumor‐derived, having their origin in other organs and cell types. Hence, liquid biopsies provide a source of direct tumor cell‐derived biomolecules and indirect nontumor‐derived surrogate markers that circulate in body fluids or are taken up by circulating peripheral blood cells. The capacity of platelets to take up proteins and nucleic acids and alter their megakaryocyte‐derived transcripts and proteins in response to external signals makes them one of the richest liquid biopsy biosources. Platelets are the second most abundant cell type in peripheral blood and are routinely isolated through well‐established and fast methods in clinical diagnostics but their value as a source of cancer biomarkers is relatively recent. Platelets do not have a nucleus but have a functional spliceosome and protein translation machinery, to process RNA transcripts. Platelets emerge as important repositories of potential cancer biomarkers, including several types of RNAs (mRNA, miRNA, circRNA, lncRNA, and mitochondrial RNA) and proteins, and several preclinical studies have highlighted their potential as a liquid biopsy source for detecting various types and stages of cancer. Here, we address the usability of platelets as a liquid biopsy for the detection of cancer. We describe several studies that support the use of platelet biomarkers in cancer diagnostics and discuss what is still lacking for their implementation into the clinic.

## Graphical abstract

Blood platelets in circulation are important repositories of potential cancer biomarkers, including several types of RNAs and proteins, which make them a rich biosource for liquid biopsy. Platelets are routinely isolated through well‐established and fast methods in clinical diagnostics. Using multiple techniques, several preclinical studies have highlighted the potential of platelet biomarkers in detecting various types and stages of cancer.

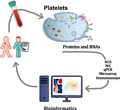

**Abbreviations**
ACC: accuracy
ALK: anaplastic lymphoma kinase
AUC: area under the curve
bFGF: basic fibroblast growth factor
BrCa: breast cancer
cfDNA: cell‐free DNA
CI: confidence interval
circRNA: circular RNA
CRC: colorectal cancer
CRPC: castration‐resistant prostate cancer
CTCs: circulating tumor cells
ddPCR: droplet Digital PCR
dsDNA: double‐stranded DNA
EGFR: epidermal growth factor receptor
EVs: extracellular vesicles
FFPE: formalin‐fixed paraffin‐embedded
FISH: fluorescence in situ hybridization
GBM: glioblastoma
HBC: hepatobiliary cancer
HD: healthy donor
IFS: incremental feature‐selection
ITGA2B: integrin alpha 2b
LGG: lower‐grade glioma
lncRNA: long noncoding RNA
LOOCV: leave‐one‐out cross‐validation
MET: mesenchymal–epithelial transition
MHC‐I: major histocompatibility complex class I
miRNA: microRNA
mRNA: messenger RNA
MS: mass spectrometry
NIPT: noninvasive prenatal test
NK: natural‐killer
NSCLC: non‐small‐cell lung cancer
PAAD: pancreatic cancer
PCa: prostate cancer
PCR: polymerase chain reaction
PDGF: platelet‐derived growth factor
PEVs: platelet‐derived extracellular vesicles
PF4: platelet factor 4
PLS‐DA: partial least squares discriminant analysis
PMPs: platelet microparticles
PSO: particle‐swarm optimization
RT–qPCR: real‐time quantitative PCR
SCLC: small‐cell lung cancer
ssDNA: single‐stranded DNA
SVM: support vector machine
TEPs: tumor‐educated platelets
TKI: tyrosine kinase inhibitor
TSP‐1: thrombospondin‐1
VEGF: vascular endothelial growth factor

## 1 Introduction

Currently, cancer is primarily detected via imaging methods and confirmed via tissue biopsies, which also support clinical decisions on treatment options. Tissue biopsies can complement imaging, giving precious information on the characteristics of the tumor, such as specific mutational signatures that show resistance to some therapies [1, 2, 3]. However, tissue sampling is invasive, often unpleasant, and involves high risk for the patient. Some tumor locations are not even easily accessible for tissue biopsy, as, for example, the brain. Since the tissue mostly is obtained at the initial sampling, it only allows for the analysis of a tumor section removed at that specific time‐point (e.g., snapshot) and can miss intratumoral heterogeneity [4]. Several types of analytes and biomarkers are released from the tumor into the bloodstream and other bodily fluids [5]. Sampling of biofluids and the analysis of their content can provide ample information on the molecular characteristics from the primary tumor but also from distant metastatic sites. The collection of liquid biopsy samples is far less invasive than tissue sampling, can be repeated several times during follow‐up, and can give a more systemic and complete representation of the disease. Liquid biopsies include circulating tumor cells (CTCs) [6, 7, 8], cell‐free DNA (cfDNA) [9, 10, 11], extracellular vesicles (EVs) [12, 13], and more recently, tumor‐educated platelets (TEPs) [14]. All these biosources present in the blood are considered powerful reservoirs of cancer biomarkers. Hence, different types of liquid biopsy biosources can be considered, or combinations thereof.

One of the emerging biosources with vast potential in liquid biopsies is the blood platelets (thrombocytes) [15, 16]. Platelets are anucleated cell fragments that originate from large progenitor cells, the megakaryocytes [17]. Platelets released into the bloodstream are the second most abundant cell type in peripheral blood. Platelets have a relatively short lifespan in the bloodstream, ranging from 8 to 11 days [18], subsequently being degraded in the spleen. Since platelets were first described in 1881 [19], they have been intensively studied and are known to have key roles hemostasis, thrombosis, immunity, inflammation, and cancer metastasis [20]. Platelets contain bioactive molecules, such as growth factors, chemokines, and cytokines, which are all key players in the formation of ‘early metastatic niches’ and metastatic progression. For instance, the recruitment of granulocytes to the metastatic niche may not be primarily due to tumor cell‐derived signals but rather to platelet‐derived CXCL5/7 chemokines [21].

Platelet collection and analysis are often included in the routine clinical diagnostics tests (e.g., the platelet count test). The ideal platelet count range is 150 to 450 × 10^9^ L^−1^ in most healthy people [22], and this number can change due to several (patho)physiological factors and in the presence of diseases such as cancer [23]. It has been shown that the presence of cancer affects not only platelet count, volume, and activation status, but also the platelet‐derived proteins and the RNA content.

The aim of this review was to describe the potential biomarkers in platelets and the usability of platelets as a liquid biopsy for the detection of cancer. We describe several studies that support the use of platelet proteins and RNAs as biomarkers in cancer diagnostics and discuss what is still lacking for their implementation into the clinic.

## 2 Platelets and their crosstalk with other liquid biopsy biosources

### 2.1 Tumor‐educated platelets

**Fig. 1:**
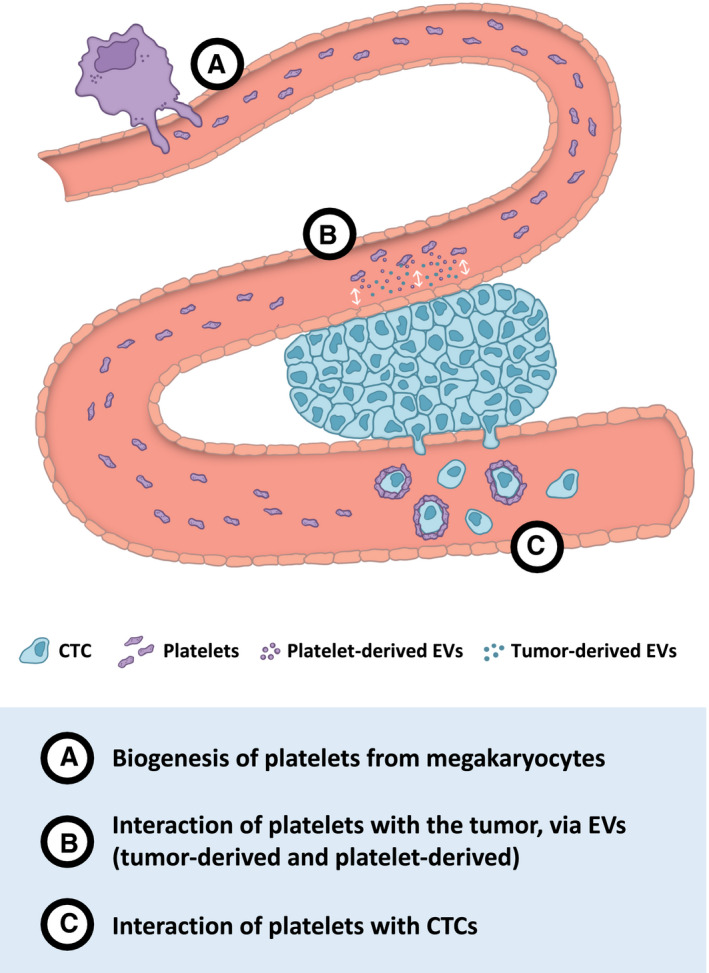
Platelets in circulation: biogenesis, interaction with CTCs, and crosstalk through EVs. Platelets originate from megakaryocytes and are released into the bloodstream (A). During their short lifespan in circulation, platelets are exposed to several interactions with other cells and the tumor microenvironment (TME). Other than the direct interaction of platelets with tumor cells, this crosstalk can occur also via extracellular vesicles (EVs). Both cancer cells and platelets release EVs, named, respectively, tumor‐derived EVs and platelet‐derived EVs (B). Circulating tumor cells (CTCs) may also activate and educate platelets. Platelets may also contribute to CTC survival, helping them escape from immune surveillance. Platelets also promote cell adhesion, arrest in vasculature and vascular permeability, facilitating the metastatic process (C).

Platelets are considered important repositories of potential RNA biomarkers: messenger RNA (mRNA), circular RNA (circRNA), long noncoding RNA (lncRNA), and mitochondrial RNA; and they have a functional spliceosome and a protein translation machinery to process the RNA transcripts [24]. Following interaction with tumor cells or tumor‐associated biomolecules, platelets can alter their RNA profile, and this process gives rise to the so‐called tumor‐educated platelets (TEPs) [25, 26, 27]. Although lacking a nucleus, platelets are equipped with RNA processing machinery, such as pre‐mRNA splicing, pre‐microRNA (miRNA) processing, and mRNA translation [24]. Most RNA content of platelets originates from their parental megakaryocytes Fig. 1A). However, platelets can also take up and store tumor‐derived RNAs, both in peripheral blood and at the tumor microenvironment Fig. 1B,C). Platelets can directly ingest circulating mRNA [26], and external signals can stimulate platelet activation and induce specific splice variants of pre‐mRNAs, giving rise to unique mRNA profiles with potential applicability in cancer diagnostics [14, 24, 28, 29, 30].

### 2.2 Interaction of TEPs with EVs and the platelet‐derived vesicles

Platelets crosstalk with tumor cells can occur directly, or via tumor‐ and platelet‐derived EVs Fig. 1B). EVs can originate from various cell types, and their content may vary depending on pathophysiological conditions. EVs derived from cells such as leukocytes, erythrocytes, megakaryocyte, platelets, and tumor cells can be detected in the circulation and are potential sources of liquid biopsies for cancer diagnostics [31]. Platelets can sequester these EVs that may contain tumor‐specific RNA [26, 32].

Depending on their biogenesis and size, the platelet‐derived EVs can have different designations. Platelet microparticles (PMPs) are a type of platelet‐derived extracellular vesicles (PEVs) and the most abundant population of EVs present in the blood [33], accounting for about 70–90% of all EVs [34]. In 1967, Peter Wolf was the first to identify these small procoagulant structures deriving from activated blood platelets and named them ‘platelet dust’ [35]. During vesiculation, part of the cytoplasm and membrane of the platelet is budding, resulting in the origination of the EVs. The cargo of the PEVs is vast and is reported to contain cytokines, functional enzymes, mRNA, and noncoding RNA, all originating from the platelet interior [36]. Platelets contain an average of four mitochondria. It has been reported that during the genesis of the PEVs, some of these mitochondria may even be encapsulated in the larger vesicles [37]. A proteomic study showed that indeed larger microparticles contained mitochondrial proteins whereas the smaller microparticles did not [37]. As for many types of EVs, PEV populations still lack a broad consensus on nomenclature. This can be achieved with further research in order to better characterize the populations originated from platelets or megakaryocytes. Although more studies are required to improve our understanding of the crosstalk between tumor cells, platelets, and their vesicles, the analysis of PEVs and their cargo may aid the discovery of potential cancer biomarkers which in parallel with the tumor‐derived EVs and TEPs may provide better diagnostics tools.

### 2.3 Interaction of TEPs with CTCs

Circulating tumor cells are one of the most commonly used biosources in the liquid biopsy field. In the bloodstream, the CTCs interact closely with other cells such as neutrophils, macrophages, and platelets. This interaction in the blood is considered crucial for the development of metastasis and may enable the identification of potential novel therapeutic targets [38, 39]. CTCs can activate and educate platelets. Platelets can ingest mRNA from cancer cells, triggering a possible modification in the platelet transcriptome that resembles tumor profile [38].

Conversely, platelets can contribute to CTC survival, helping them escape from immune surveillance [40, 41] and from the high shear forces in the blood circulation [42]. Platelets adhere to and physically protect CTCs, by surrounding CTCs with a cell fibrin‐platelet aggregate [41, 43, 44] Fig. 1C). In addition, major histocompatibility complex class I (MHC‐I) molecules can be transferred from platelets to CTCs, which then escape recognition by natural‐killer (NK) cells CTCs [45]. Platelets also promote cell adhesion, arrest in the vasculature, and vascular permeability, facilitating the metastatic process [39, 46, 47, 48, 49]. The molecular processes involved in the interaction between platelets and CTCs are still not completely known. Complementary studies of platelets, CTCs, and EVs need to be performed in order to understand more about this crosstalk. Better understanding of biological interactions and underlying processes may provide more insight on the value of combining different biosources in liquid biopsy assays.

## 3 Biomarkers from platelets

### 3.1 mRNA, circRNA, and lncRNA in platelets

**Fig. 2:**
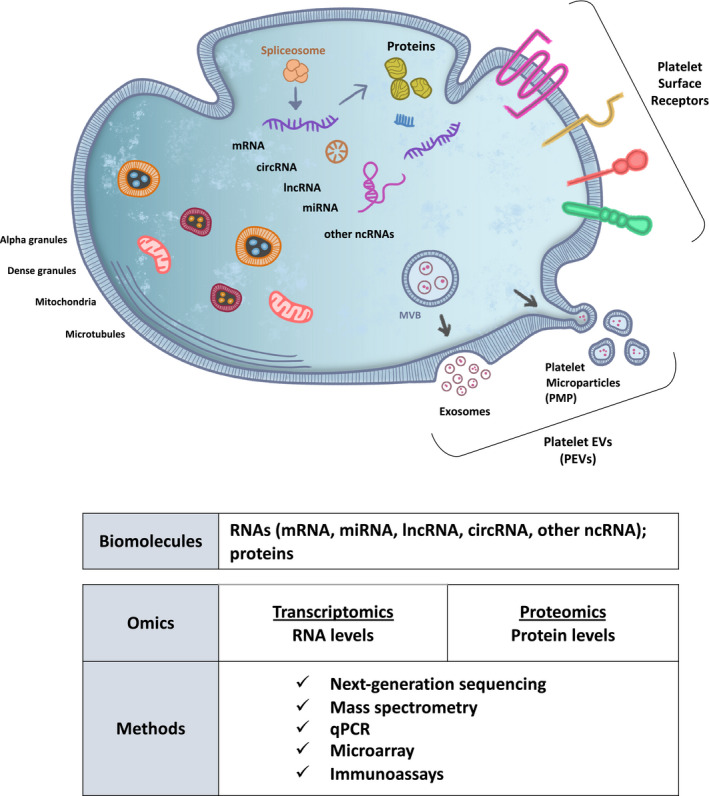
Composition, biomarkers, and omics analysis of platelets. Schematic representation of molecular components of platelets. Platelets are considered important repositories of diagnostic biomarkers, among them are the RNA biomarkers: mRNAs, miRNAs, circRNAs, lncRNAs, and other ncRNAs. Platelets do not have a nucleus but have a functional spliceosome and protein translation machinery, to process RNA transcripts. As most cells, platelets release extracellular vesicles (EVs). The platelet‐derived extracellular vesicles (PEVs) can have different designations, based on their biogenesis and size. Platelet microparticles (PMPs) are a type of PEVs and the most abundant population of EVs present in the blood, accounting for about 70% to 90% of all EVs. The PMPs are released via budding of the platelet membrane. Exosomes are also released by platelets, they are smaller and release out of the platelet upon fusion of an intermediate endocytic compartment, the multivesicular body (MVB), with the plasma membrane of the platelet. The PEVs carry components from the lumen of the platelet where they originated. Platelets have granules that release their content to the external upon activation. Dense granules contain phosphates, purines, and bioactive amines; alpha granules contain many soluble mediators that promote inflammation and coagulation. Platelets contain also a large set of membrane surface receptors that participate in the platelet activation process and act as adhesion molecules. The platelet receptors are integrins (β1, β2, and β3), leucine‐rich repeats receptors (GPIb‐IX‐V, TLR, and MMP); selectin s (P‐selectin, CLEC‐2, and CD72); tetraspanins (CD63, CD9, and CD53); transmembrane receptors (ADP and thrombin); prostaglandin receptors (thromboxane, PGI2, PGD2, and PGE2); lipid receptors (PAF and LPL‐R); immunoglobulin superfamily receptors (GPVI, CD32, CD23, JAM, PECAM‐1, CD31, and TLT‐1); tyrosine kinase receptors (c‐mpl, CD110, Leptin, Tie‐1, insulin, and PDGF); miscellaneous platelet membrane receptors (5‐HT2A, CD36, C1qR, LAMP‐1, CD107a; LAMP‐2, CD107b, and CD40L).

When in contact with tumor cells or tumor‐associated proteins, the platelets can be ‘educated’ and alter their RNA profile [26, 27]. The platelets are considered important repositories of potential RNA biomarkers (mRNA, miRNAs, circRNA, lncRNA, and mitochondrial RNA) [50], and they have a functional spliceosome and a protein translation machinery to process the RNA transcripts (Fig. 2). Several studies are focusing on mRNA platelet profiling using sequencing methods, to detect and monitor cancer patients [25, 51]. CircRNAs are another type of RNA which is also significantly enriched in platelets, where they most commonly are generated from exons of protein‐coding genes through back‐splicing. Several distinct circRNAs were identified in platelets by selectively removing linear transcripts. Further studies are needed to gain a more detailed insight into the biogenesis and function of circRNAs in platelets [52, 53]. A study also showed that in prostate cancer (PCa), the measurement of long non‐coding (lncRNAs) [54] and microRNAs (miRNAs) [55] in blood offers promising prospects to serve as PCa biomarker, since both types of RNA are often tumor‐specific and relatively easy to detect [56]. Such RNAs can be taken up by platelets, hence hypothetically also detected in the isolated platelets. Further investigations are required to pinpoint which fractions of the blood contain specific RNA types and their potential as clinical utility.

### 3.2 Proteins in platelets

The protein content of platelets can consist of megakaryocyte‐derived proteins, endocytosed proteins, and proteins translated into individual platelets. Therefore, it is important to determine which platelet proteins are truly tumor‐derived and which proteins may fluctuate due to tumor‐independent processes. Besides the previously described uptake of RNA molecules, platelets can also sequester proteins [57]. Platelets contain secretory granules that release their content to the external upon activation [20]. Alpha granules contain many soluble mediators that promote inflammation and coagulation [58]. Additionally, platelets contain also a vast set of membrane surface receptors that participate in the platelet activation process and act as adhesion molecules [59]. After isolation of platelets from whole blood, proteins are made accessible by platelet lysis, followed by enzymatic or chemical protein digestion [60]. The activation status and the analysis of the platelet surface proteome can be a source of potential cancer biomarkers. Over the past years, advances in mass spectrometry‐based methods have greatly supported proteomic studies on revealing numerous proteins from platelets [60, 61, 62, 63]. For example, one study identified 1507 proteins in platelets, 190 of which membrane proteins, and 262 phosphoproteins [64]. Many platelet proteomic studies can be found in the PlateletWeb [64]. This platform is a comprehensive human platelet repository for systems biologic analysis of platelets, in the functional context of integrated networks. Functional, drug, and pathway associations can be analyzed and studies included count with more than 5000 proteins [64].

## 4 Tumor‐educated platelet tests

A range of cancer biomarkers can be found in tumor‐educated platelets. Several studies have shown the importance of such biomarkers using different techniques to detect and monitor numerous cancer types Table 1.

**Table 1:** Platelets in liquid biopsies: List of potential biomarkers and tests.

Cancer type	Cancer stage	Test	Platelets biomarkers	Cohort	Techniques	Reference
Pan‐cancer: Non‐small‐cell lung carcinoma (NSCLC), colorectal cancer (CRC), glioblastoma (GBM), pancreatic cancer (PAAD), hepatobiliary cancer (HBC) and breast cancer (BrCa)	Early‐ and late‐stage	Accuracy = 96%	mRNA	*n* = 283: Patients (*n* = 228) and healthy individuals (*n* = 55)	RNA‐seq, multiclass Support vector machine (SVM)‐based classification	[25]
Lung and pancreatic	Early‐ and late‐stage	AUC = 88.7% (Lung, not including the smoking variable) AUC = 94.5% (Lung‐ including the smoking variable) AUC = 82.7% (Pancreas) After internal validation, resulting in optimism‐corrected AUC of 86.8% (Lung) and 80.8% (Pancreas)	Platelet count, volume, protein content, activation status (and smoking)	Patients with lung cancer (*n* = 86), head of pancreas cancer (*n* = 42). Healthy sex‐ and age‐matched controls (*n* = 92)	Multivariable diagnostic models	[66]
Lung and pancreatic	Early‐stage	4384 unique proteins of which 85 were significantly changed in early‐stage cancer compared to controls (criteria Fc > 1.5 and *P* < 0.05)	Proteome	Patients (*n* = 12): early‐stage lung (*n* = 8) and head of pancreas cancer (*n* = 4) Healthy sex‐ and age‐matched controls (*n* = 11)	Mass spectrometry	[72]
Lung (NSCLC)	Late‐stage	Accuracy = 88%; AUC = 0.94; CI = 95%, 0.92–0.96; *P* < 0.001	Selection of RNA biomarker panels from platelets	*n* = 779: Patients (*n* = 402) and Control individuals with no known cancer, but not excluding individuals with inflammatory diseases (*n* = 377)	RNA‐seq; Particle‐swarm optimization (PSO)‐enhanced algorithms	[51]
Lung (NSCLC)	Early‐ and late‐stage	Sensitivity = 0.925, Specificity = 0.827, Accuracy = 0.889	48‐biomarker genes panel (SVM and LOOC); WASF1, PRKAB2, RSRC1, PDHB, TPM2, MYL9, and PPP1R12C (Network analysis)	NSCLC patients (*n* = 402) and healthy controls (*n* = 231)	RNA‐seq; Advanced minimal redundancy, maximal‐relevance, and incremental feature‐selection (IFS); Support vector machine (SVM) classifier; LOOCV	[70]
Lung (NSCLC)	Early‐stage (I–II)	Test cohort: Accuracy AUC of 0.922 [95% confidence interval (CI), 0.892–0.952], Sensitivity = 92.8% Specificity = 78.6% Validation cohort: AUC = 0.888, Sensitivity = 91.2%, Specificity = 56.5%	ITGA2B	RNA‐seq: Patients (*n* = 9 and healthy controls (*n* = 8). Polymerase chain reaction (PCR): Patients with NSCLC (*n* = 243), Healthy Controls (*n* = 150), and Patients with benign pulmonary nodules (*n* = 141)	RNA‐seq, quantitative real‐time PCR (qPCR) and Droplet Digital PCR (ddPCR)	[76]
Lung (NSCLC)	Late‐stage (III–IV)	Sensitivity = 78.8%, Specificity = 89.3%, Accuracy = 83.6%	RNA isolated from platelets and from plasma. (EML4)‐ALK	ALK‐Fluorescence in situ hybridization (FISH) Positive (*n* = 33) and ALK‐FISH Negative (*n* = 28) Patients	RT–PCR	[77]
Lung	Early‐ and late‐stage	Area under the curve (AUC) = 0.734 AUC = 0.787 (early‐stage) AUC = 0.825 (females)	mRNA: MAX, MTURN, and HLA‐B	Lung cancer patients (*n* = 225) and Healthy donors (*n* = 185)	Microarray; qPCR	[79]
Lung	Late‐stage	N/A	cfRNA. PF4 (possible interactions with: PF4 and CLU, CCL5, TGFB1, SRGN, and SPARC)	Small‐cell lung cancer (SCLC) Patients (*n* = 10), NSCLC Patients (*n* = 10) and Healthy volunteers (*n* = 4)	NanoString nCounter	[71]
Glioblastoma (GBM) and pancreatic	Late‐stage	Sensitivity = 80%, Specificity = 96%	EGFRvIII and PCA3	Glioma Patients (*n* = 8) and Control Subjects (*n* = 12)	RT–PCR	[26]
Glioblastoma	Late‐stage	Accuracy = 80%, AUC = 0.81, 95% CI, 0.74–0.89; *P* < 0.001 (glioblastoma, multiple sclerosis and brain metastasis) Accuracy = 95%, AUC = 0.97, 95% CI, 0.95–0.99; *P* < 0.001 (glioblastoma and asymptomatic healthy controls) Accuracy = 85%, AUC = 0.86, 95% CI, 0.70–1.00; *P* < 0.012 (false‐positive progression and true progression)	mRNA	Validation series: *n* = 157 (glioblastoma, multiple sclerosis and brain metastasis) Validation series: *n* = 347 (glioblastoma and asymptomatic healthy controls) Validation series, *n* = 20 (false‐positive progression and true progression)	RNA‐seq; PSO‐enhanced algorithms	[68]
Prostate‐ castration‐resistant prostate cancer (CRPC)	N/A	AUC = 0.76, *P* < 0.05 (PSA response) AUC = 0.84, *P* < 0.01 (three‐gene panel)	KLK3, FOLH1, NPY	Patients (*n* = 50): receiving docetaxel (*n* = 24) and abiraterone (*n* = 26) therapy. Healthy controls (*n* = 15)	Digital PCR	[65]
Ovarian	Late‐stage (III–IV)	Sensitivity = 96% Specificity = 88%	Proteome	Benign ovarian lesions (*n* = 16) and ovarian cancer, FIGO stages III–IV (*n* = 20)	Partial least squares discriminant analysis (PLS‐DA)	[73]
	Early‐stage (I–II)	Sensitivity = 83%, Specificity = 76%, cut‐off > 0.5. AUC = 0.831, *P* < 0.0001		Benign adnexal lesions (*n* = 28), ovarian cancer FIGO stages I–II (*n* = 8), and ovarian cancer, FIGO stages III–IV (*n* = 32)	Western blot; PLS‐DA	
	Late‐stage (III–IV)	Sensitivity = 70% Specificity = 83%		Patients with ovarian cancer, FIGO stages III–IV (*n* = 30), and benign adnexal control lesions (*n* = 29)	DigiWest; PLS‐DA	
Colorectal	Early‐ and late‐stage	AUC = 0.893, *P* < 0.0001	Proteome. VEGF, PF4 and PDGF	Patients (*n* = 35) and age‐ matched healthy controls (*n* = 84)	ELISA	[74]
Breast	Early‐ and late‐stage	AUC = 0.9705 [95% confidence interval (CI): 0.9494–0.9823]	TPM3 mRNA	Patients (*n* = 549) and age‐matched healthy volunteers (*n* = 154)	RNA‐seq, qRT–PCR, western blot	[78]
Lower‐grade glioma (LGG)	N/A	Accuracy = 88% (Validation set only; *n* = 24 samples, AUC = 0.86, 95% CI = 0.70–1.00)	mRNA	Patients (*n* = 39), Healthy donors (*n* = 41)	RNA‐seq; PSO‐enhanced algorithms	[67]
Sarcoma	Early‐ and late‐stage	Accuracy = 87% (Validation set only; *n* = 53 samples, AUC = 0.93, 95% CI: 0.86–1)	mRNA	Patients with active disease (*n* = 57), former sarcoma patients, cancer free for ≥ 3 years (*n* = 38) and healthy donors (*n* = 65)	RNA‐seq; PSO‐enhanced algorithms	[69]

### 4.1 NGS analyses and the biocomputational tools to identify TEP biomarkers

In a prospective study, changes in platelet count, volume, protein content, and activation status of patients with lung cancer or head of pancreas cancer were detected, when compared to healthy sex‐ and age‐matched controls. The diagnostic model developed for lung cancer discriminated between patients and controls [area under the curve (AUC) 88.7%]. The addition of smoking as a variable significantly increased the AUC of the model to 94.5%. The diagnostic model for pancreas cancer also performed well (AUC 82.7%). Both models were internally validated, resulting in corrected AUCs of 86.8% and 80.8%, respectively [66].

In another study, a microarray analysis of isolated platelets was used to discover an RNA signature that could distinguish patients with glioblastoma from healthy controls. In addition, 17 out of the top 30 altered transcripts were also found in the mRNA sequencing data from TEPs of glioblastoma (GBM) patients. Among which, four of the most significantly differentially expressed genes were as follows: WFDC1, FKBP5, IL1R2, and TPCN1 [26].

Another study could discriminate between patients with lung cancer patients and healthy individuals with 96% accuracy using the thromboSeq pipeline involving the mRNA sequencing of 283 TEP samples [67]. The algorithm predicted the presence of the mesenchymal–epithelial transition (MET) amplification, epidermal growth factor receptor (EGFR) mutations, and KRAS mutations in the tumors [25]. The indirect detection of tumor‐driving mutations via platelet RNA surrogate signatures reflects the potential use of TEPs to predict the therapeutic responses of cancer patients [14]. Particle‐swarm optimization (PSO)‐enhanced algorithms enabled the efficient selection of RNA biomarker panels from platelet RNA sequencing libraries (*n* = 779). This resulted in accurate TEP‐based detection of mainly late‐stage non‐small‐cell lung cancer (NSCLC) (*n* = 518 late‐stage validation cohort, accuracy, 88%, AUC, 0.94, 95% CI, 0.92–0.96, *P* < 0.001), independent of age of the individuals, smoking habits, whole‐blood storage time, and diverse inflammatory conditions. PSO enables the selection from mRNA TEPs data, the gene panels more suitable in that cohort, to diagnose cancer [51].

Furthermore, the PSO‐enhanced thromboSeq approach was also applied for the analysis of brain malignancy datasets. One study used a dataset including platelet RNA‐seq data from 39 lower‐grade glioma (LGG) patients and 41 healthy donors (HD). The classification of HD and LGG samples in the validation series was done with an accuracy of 88% and AUC = 0.86 [95% confidence interval (CI) = 0.70–1.00, *n* = 24] [67]

Another study using also thromboSeq analyzed primary glioblastoma patients, multiple sclerosis, and brain metastasis. This validation had an accuracy (ACC) of 80% (*n* = 157; AUC, 0.81 [95% CI, 0.74–0.89; *P* < 0.001]). A second analysis was done comparing the glioblastoma patients and asymptomatic controls in an independent validation cohort of 347 samples, performing with 95% accuracy and an AUC of 0.97 (95% CI, 0.95–0.99; *P* < 0.001). Importantly, further development of the digitalSWARM algorithm as a tool to monitor GBM progression provided the demonstration that the TEP score represents tumor behavior and could be used to distinguish false‐positive progression from true progression (validation series, *n* = 20; accuracy, 85%; AUC, 0.86 [95% CI, 0.70–1.00; *P* < 0.012]). Although the initial analysis has provided promising proof‐of‐concept results, more samples should be included in future studies to make the prediction algorithm more robust, especially for the detection of false‐positive progression [68].

A recent study analyzed RNA‐seq data with the thromboSeq pipeline, including PSO‐enhanced analysis and ANOVA statistics [67] for the detection of sarcoma. Samples from 57 patients with active sarcoma were tested versus the control group, which included 38 former patients (sarcoma‐free for ≥ 3 years) and 65 healthy donors. The results of the ANOVA identified unique platelet RNA expression patterns of 2647 genes (false discovery rate < 0.05) in sarcoma patients in comparison with the control group. The PSO‐enhanced diagnostic accuracy was 87% for the validation set (*n* = 53 samples, AUC of 0.93). Interestingly, the authors performed a Venn diagram analysis to access the uniqueness of the sarcoma signature obtained, comparing it with previous studies in NSCLC [51] and in LGG [67], and concluded that a unique sarcoma signature can be selected from TEP RNA profiles [69]. Altogether, these studies suggest that the data analysis based on the PSO‐enhanced algorithms approach, such as thromboSeq, may also benefit the optimization of diagnostics readout not just for TEPs but also of other liquid biopsy biosources.

Using RNA‐seq data of TEPs from patients with NSCLC and healthy controls, a total of 48‐biomarker panel was selected [70]. A support vector machine (SVM) classifier based on the 48‐biomarker panel accurately predicted NSCLC with leave‐one‐out cross‐validation (LOOCV), with 0.925 sensitivity, 0.827 specificity, and 0.889 accuracy. Network analysis of the 48 transcripts revealed that the WASF1 actin cytoskeleton module, the PRKAB2 kinase module, the RSRC1 ribosomal protein module, the PDHB carbohydrate‐metabolism module, and three intermodule hubs (TPM2, MYL9, and PPP1R12C) might play important roles in NSCLC tumorigenesis and progression [70].

New technologies are also emerging, and the RNA biomarker panel approach for testing is starting to be explored. Using a technology based on hybridization techniques, the NanoString, a study analyzed CTCs and plasma cfRNA from patients with metastatic lung cancer, and the objective was to identify potential tumor‐associated biomarkers [71]. NanoString analysis based on CTC and plasma cfRNA data highlighted an intriguing platelet factor 4 (PF4)‐centric network, including several possible interactions between PF4 and CLU, CCL5, TGFB1, SRGN, and SPARC. This model still needs careful validation through focused clinical and laboratory‐based studies [71].

### 4.2 Proteomics to identify TEP biomarkers

Analysis of the platelet proteome of patients with early‐stage (stage I–II) lung and pancreas cancer demonstrated that the platelet proteome of patients is significantly different from that of healthy controls matched for sex and age [72]. Additionally, the platelet proteome appeared to normalize after surgical resection of the tumor, suggesting that the platelet proteome may be a powerful biomarker for early‐stage cancer detection and disease status monitoring [72]. In ovarian cancer patients, a group of platelet protein biomarker candidates that can distinguish between ovarian cancer cases as compared to benign adnexal lesions was identified using PLS‐DA of platelet protein expression in 2D gels [73]. The results suggested differences between the International Federation of Gynaecology and Obstetrics (FIGO) stages III–IV of ovarian cancer, compared to benign adnexal lesions with a sensitivity of 96% and a specificity of 88%. A PLS‐DA‐based model correctly predicted 7 out of 8 cases of FIGO stages I–II of ovarian cancer after verification by western blot with a sensitivity of 83% and specificity of 76% (AUC 0.831, *P* < 0.0001). Validation on an independent set of samples by DigiWest with PLS‐DA differentiated benign adnexal lesions and ovarian cancer, FIGO stages III–IV, with a sensitivity of 70% and a specificity of 83% [73]. Another study used ELISAs with platelet and plasma samples from 35 patients with colon cancer and 84 age‐matched healthy individuals to compare the levels of, vascular endothelial growth factor (VEGF), basic fibroblast growth factor (bFGF), platelet‐derived growth factor (PDGF), PF4, and thrombospondin‐1 (TSP‐1). Statistically significant differences were found in the median levels of VEGF, PF4, and PDGF in platelets of patients with colon cancer compared with healthy individuals. Multivariable logistic regression analysis indicated that PDGF, PF4, and VEGF were independent predictors of colorectal carcinoma and as a set provided statistically significant discrimination (AUC 0.893, *P* < 0.0001) [74].

### 4.3 Testing biomarker panels with RT–qPCR

Several of the studies mentioned above have demonstrated the huge potential of platelets as a source of liquid biopsies for detecting cancer, even considering the restricted number of biomarkers that have been derived from platelet samples of patients with cancer. The most common method used to detect RNA biomarkers in platelets is real‐time quantitative PCR (RT–qPCR). Below we discuss some potential platelet mRNA biomarkers in various types and stages of cancer.

EGFRvIII is a deletion mutant of *EGFR* [75], and the EGFRvIII RNA transcript is present in 30% of glioblastoma tumors. RT–PCR analysis of platelets from patients with glioblastoma detected the EGFRvIII RNA transcript with a sensitivity of 80% and a specificity of 96% [26]. By testing isolated platelets with RT–PCR for a single RNA biomarker, integrin alpha 2b (ITGA2B), it was possible to differentiate among patients with stage I NSCLC, individuals with benign lung nodules, and healthy controls [76]. The diagnostic accuracy of ITGA2B testing in platelet samples was AUC 0.922, with a sensitivity of 92.8%, and specificity of 78.6% in the test cohort [76]. In addition, an AUC of 0.888 was achieved in the validation cohort for NSCLC, with a sensitivity of 91.2% and specificity of 56.5% [76].

Another study evaluated mRNAs transcribed by anaplastic lymphoma kinase (*ALK)*‐mutated genes as biomarkers in NSCLC. RT–PCR was used to detect the EML4‐*ALK* rearrangement in RNA extracted from formalin‐fixed paraffin‐embedded (FFPE) tissues, or RNA isolated from the plasma, or platelets of patients with NSCLC. In the validation cohort, RT–PCR analyses of liquid biopsies (plasma and platelets) had higher sensitivity (78.8%), specificity (89.3%), and accuracy (83.6%) as compared with RT–PCR analysis of FFPE tissues [77]. Patients tested at least 6 months after diagnosis showed high rates of ALK rearrangements in the plasma (85.7%) and in platelets (87.0%) [77]. Within a smaller group of 26 of the patients that had been previously treated with the ALK tyrosine kinase inhibitor (TKI) crizotinib, the subgroup that tested positive for *ALK* rearrangements within the platelet fraction showed a longer median duration of treatment (7.2 versus 1.5 months), longer median progression‐free survival (5.7 versus 1.7 months), a higher overall response rate (70.6% versus 11.1%), and a higher disease control rate (88.2% versus 44.4%) than the subgroup that tested negative for *ALK* rearrangements. Due to a limited number of samples (*n* = 26) included in the study, a multivariate analysis in a larger sample group is required to further confirm the value of platelet analysis in therapy selection for ALK‐positive patients [77]. Platelets may therefore also be useful in predicting treatment outcomes of ALK inhibitor.

The platelet‐derived TPM3 mRNA has been shown to be delivered into the tumor through microvesicles and potentiate the migrative phenotype of breast cancer cells. Upregulation of TPM3 mRNA in platelets significantly correlated with metastasis in patients with breast cancer. Hence, platelet‐derived TPM3 mRNA may be a suitable biomarker for early diagnosis of metastatic breast cancer [78].

A panel of three‐platelet mRNAs, namely MAX, MTURN, and HLA‐B, was selected by microarray and validated by qRT–PCR as a biomarker in lung cancer. Detection of this panel was significantly higher in patients with lung cancer, including early‐stage patients, as compared with healthy controls (AUC of 0.734 and 0.787, respectively) [79]. Of the three mRNAs, platelet MTURN mRNA showed high diagnostic efficiency in female patients with lung cancer (AUC 0.825). More importantly, this panel was associated with chemotherapeutic effects, as low levels of these three‐platelet mRNAs were correlated with ‘favorable’ first chemotherapy response in these patients [79].

## 5 Translation of TEP biomarkers into the clinic

While the platelet transcriptome is dynamically altered in disease states [80, 81, 82], in the absence of disease platelet gene expression remains remarkably stable [83]. One of the challenges in using TEPs as liquid biopsy for cancer diagnostics is to understand which transcriptomic profiles are specifically associated with, for example, inflammation, other noncancerous processes, and therapeutics (anticoagulants and other pharmacological treatments).

Currently, TEP‐derived proteins and RNA have emerged as promising cancer biomarkers in several studies including a vast spectrum of cancers, such as ovarian cancer, prostate cancer, lung cancer, colorectal cancer, glioblastoma, pancreatic cancer, hepatobiliary cancer, and breast cancer [14, 25, 27, 65, 73, 84] (Table 1). Nilsson *et al*. described the uptake of EVs by circulating platelets. Via this mechanism, platelets can sequester EVs from cancer cells containing tumor‐specific RNA [26, 32]. Many studies have shown that EVs contain DNA namely single‐stranded DNA (ssDNA), double‐stranded DNA (dsDNA), and mitochondrial DNA [85, 86, 87]. The capacity of platelets to ingest proteins and nucleic acids suggests that also cfDNA can be associated with platelets. Platelets can be considered a rich biosource in the blood for the discovery of cancer biomarkers with the potential to be used as liquid biopsy [88], and, with the advantage of easy isolation methods [89]. Platelets are already being isolated and counted in the routine of clinical diagnostic laboratories but their utility as a source of biomarkers currently remains at the level of clinical research.

Some considerations are needed when isolating platelets, especially for omics analysis, like proteomics and transcriptomics. The standard protocol to isolate platelets is based on 2‐step centrifugation of the whole blood, but the time, duration, and temperature of the centrifugation differ from different studies and laboratories [67]. During the processing, platelet activation can be an issue as it may induce the release of EVs and procoagulation factors [90, 91]. The chemical (buffers) and/or physical (agitation, centrifugation, or materials) conditions to which the platelets are exposed [92]. EDTA‐, citrate‐ or heparin‐coated tubes are used for TEP isolation as these buffers inhibit instant platelet activation and aggregation. Tubes coated with buffers designated for serum are not recommended as they induce blood clotting [67]. For the thromboSeq protocol, whole blood can be stored up to 48 h at room temperature in EDTA‐coated tubes until platelet isolation to maintain high‐quality RNA and the dominant cancer RNA signatures [67]. This relatively large window of time permits the implementation of a centralized and specialized diagnostic facility, for the processing of such samples. As implemented already for other types of liquid biopsy tests, for example, with the noninvasive prenatal test (NIPT) test using cfDNA [93, 94, 95], potential contamination by leukocytes or other blood cells may occur during TEP isolation and therefore potentially compromise the outcome of omics analysis [96], however, by using the standardized thromboSeq method and by avoiding disturbance of the buffy coat during platelet isolation significant contamination can be avoided [67].

Consensus on methods of normalization, sample collection, and processing is still required. The lack of uniformity can make it challenging to compare different preclinical and clinical studies, failing potential novel biomarkers to be clinically implemented. Cross‐validation of large cohorts of patients and the involvement of liquid biopsy biobanks can help harmonize TEP isolation protocols across different laboratories. These platforms can be coupled to already existing tissue biobanks or clinical diagnostic laboratories, existing in some hospitals. Collaborations between academia and industry are crucial for the optimization of liquid biopsy assays. Additionally, the introduction of laboratory protocols that are fully automated may overcome potential intercenter variability and random variation during the processing of samples.

Clinical trials will be crucial in the near future to validate the implementation of TEP tests in the clinical setting. Although it may seem premature to consider large clinical trial setups with the existing preclinical studies, a clinical utility of the TEPs diagnostic platform for cancer detection and ﻿monitoring of tumor progression should be further pursued performed as done for other types of biosources in liquid biopsies. For the assays to reach clinical application, simplified and standardized methodologies for platelet isolation and processing must be developed. At the moment, the majority of the TEP biomarkers are identified by genetic (transcriptomics) or mass spectrophotometric (proteomics) methodologies that are not available in basic clinical laboratories. Analysis based on omics data must be accompanied by user‐friendly analysis platforms with user‐friendly software [97, 98]. Aiming for a panel with limited genes, that can still distinguish between the two groups, can be advantageous for the step of diagnostic assay development. Overall, immuno‐based assays, RT–qPCR, ELISA, and Nanostring are methods less complex than RNA‐seq or mass spectrometry (MS) and warrant further analysis [99].

## 6 Conclusions and perspectives

While blood platelets in the clinic are strongly related to cancer, their utility as a rich source of biomarkers for cancer diagnostics warrants further prospective validation. Platelets have a vast repertoire of potential cancer biomarkers. Nonetheless, there is still a large gap between biomarker discovery and clinical validation and consequently also the clinical implementation of these tests. Anticoagulants, cancer therapies, inflammatory diseases, and other confounding factors can play a role in data processing from platelet samples [82]. Normalization of the input material is critical for downstream processing and analysis. This can be accomplished by using the platelets count and/or by quantification of material isolated from platelets [67]. Patient and control blood samples should be collected and processed under the same conditions, also to minimize handling‐related variability. Consensus on methods of normalization, sample collection, and processing is important [95]. The lack of uniformity can make it challenging to compare different preclinical and clinical studies, resulting in the failure of potential novel biomarkers to be implemented. To ensure that promising biomarkers discovered in research are tested in clinical trials and reach clinical practice, technical standardization is warranted [67]. Another critical point for the TEP tests would be to perform additional clinical utility studies. The clinical validation of liquid biopsy biomarkers is needed to assure their value in clinical practice. The clinical validation and clinical utility can help to develop strategies and identify frail points that need to be addressed in the tests currently being developed [57]. Discordance in results between proteomics and transcriptomics studies, urgency for uniformed isolation methods, and challenges in clinical implementation requires additional investigation. Nonetheless, platelets hold significant potential as liquid biopsies and warrant further research.

## Conflict of interest

TW is shareholder of GRAIL Inc. DK‐L is shareholder of Exbiome BV. MA‐F declares no conflict of interest.

## Author contributions

MA‐F wrote the paper; DK‐L and TW reviewed and edited the paper.

### Peer Review

The peer review history for this article is available at https://publons.com/publon/10.1002/1878‐0261.12859.
